# Pedicled abdominal skin flap technique for fingers salvaging and reconstruction in a complex palmar hand burn injury: A case report

**DOI:** 10.1016/j.ijscr.2023.109199

**Published:** 2023-12-26

**Authors:** Arif Tri Prasetyo, Lisa Y. Hasibuan, Muhammad Arsyad

**Affiliations:** Division of Plastic Reconstructive and Aesthetic Surgery, Department of Surgery, Faculty of Medicine, Universitas Padjadjaran, Bandung, Indonesia

**Keywords:** Full-thickness burn injury, Pedicled abdominal skin flaps

## Abstract

**Introduction and importance:**

The only treatment option for full-thickness burn injury is surgical management, either skin grafting or a skin flap. Treatment may be challenging due to the multiple procedures that need to be performed and multiple factors that can affect treatment outcomes especially to do fingers reconstruction.

**Case presentation:**

A 25-years-old man was admitted because of a burn injury on the palm of his left hand. There are waxy and leathery appearances of burn injuries on the palm and 2nd to 5th digits of the left hand and diagnosed with a full-thickness contact burn injury and compartment syndrome. The patient underwent a pedicled abdominal skin flap followed by necrotomy, flap thinning, and digit separation as a reconstruction management.

**Clinical discussion:**

Pedicled abdominal skin flap is one of the best surgical techniques available for full thickness burn injury reconstruction because it is believed to regain the closest natural-looking appearance and extremity functions. Abdominal flap as random flap is safe to be divided into small part to cover the fingers.

**Conclusion:**

Thorough examinations and appropriate management such as pedicled abdominal skin flaps are important to perform in patients with full-thickness burn injuries.

## Highlights

• Full thickness burn hand palmar injury is challenging.
• Immediate coverage is mandatory to salvage hand and finger.
• A split abdominal flap is considered safe after passing the critical period necessary for the flap's survival.

## 1 Introduction

Burn injury is a form of tissue damage or loss caused by contact with heat sources, such as fire, hot water, chemicals, electricity, and radiation. Burn injuries can be caused by direct or indirect exposure to heat. Depending upon the depth of tissue damage, burns may be classified into five main categories: superficial (epidermal), superficial partial-thickness, deep partial-thickness, full-thickness, and deeper injury [1]. Full-thickness burns destroy both layers of the skin (epidermis and dermis), penetrate deeper into underlying structures and have a white-leathery appearance. Full-thickness burns will not heal spontaneously by epithelialization; therefore, a reconstructive surgical procedure is needed to regain the epithelial structure of the burn-affected area [2].

Numerous surgical interventions are accessible for treating full-thickness burn injuries. One such reconstructive procedure is the pedicle flap. This surgical technique involves relocating a tissue flap from one part of the body to another while maintaining its original blood supply. In the case of an abdominal pedicle flap, the donor pedicle relies on vascularized areas supplied by arteries such as the superficial inferior epigastric (SIEA), superficial circumflex iliac (SCIA), and superficial external pudendal (SEPA), as well as dorsal and para-umbilical perforators (PUP). The utilization of a pedicled abdominal skin flap stands out as one of the most effective surgical methods for reconstructing full-thickness burn injuries, as it is believed to yield results that closely mimic natural appearance and restore extremity functions [3,4].

## 2 Case presentation

**Fig. 1:**
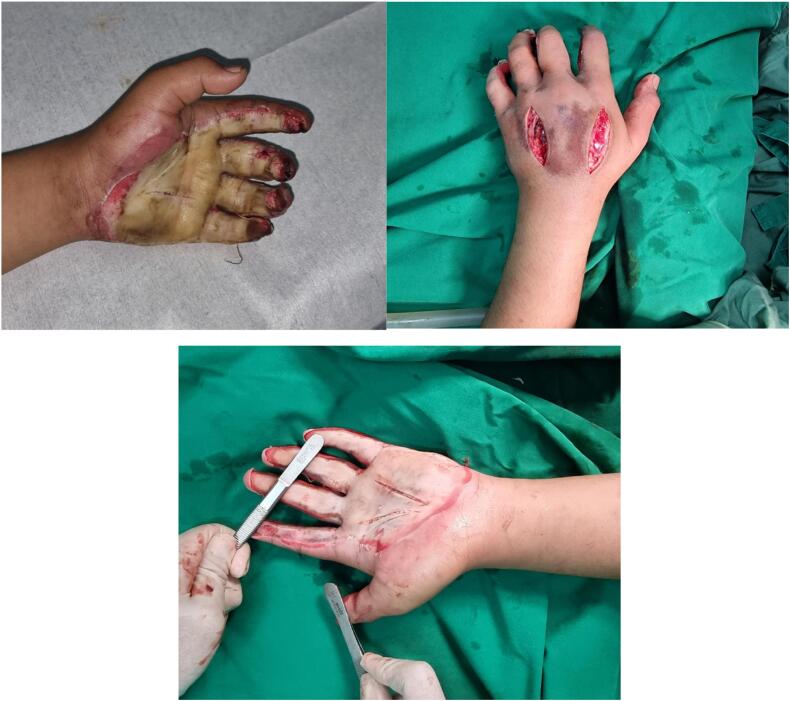
The wound demarcation and escharotomy result at first admission.

Male, 25 years old, came to Emergency Department due to an injury to the palm of his left hand. The type of injury was burn. The patient is a laborer working in a garment factory. About 1 h before admission, during his working time, patient was operating a press heating machine and accidentally pressed his own hand. Fig. 1 showed that the hand was pressed for approximately 30 s before he could undo and escape the machine pressing mechanism. Before admission, the patient did not take any medications and only spilled cold water to the injured area.

The patient presented with burn injuries localized to the palm and the second to fifth digits of the left hand. Upon examination, the patient reported pain when the affected area was touched. Additionally, the patient experienced difficulty in moving the distal part of the affected fingers. Both primary and secondary surveys yielded results within normal ranges. Based on the comprehensive evaluation, the patient was diagnosed with a full-thickness contact burn injury, affecting approximately 1 % of the Total Body Surface Area (TBSA). Additionally, compartment syndrome was identified during the examination due to the patient's reported pain, pallor in the hand, absence of a palpable pulse, which correlated with the low saturation level measured at less than 60 %. Paresthesia and paralysis were also observed in the fingers. At the emergency department, we gave the patient tetanus vaccine and analgetic injection as well as antibiotic empirically.

**Fig. 2:**
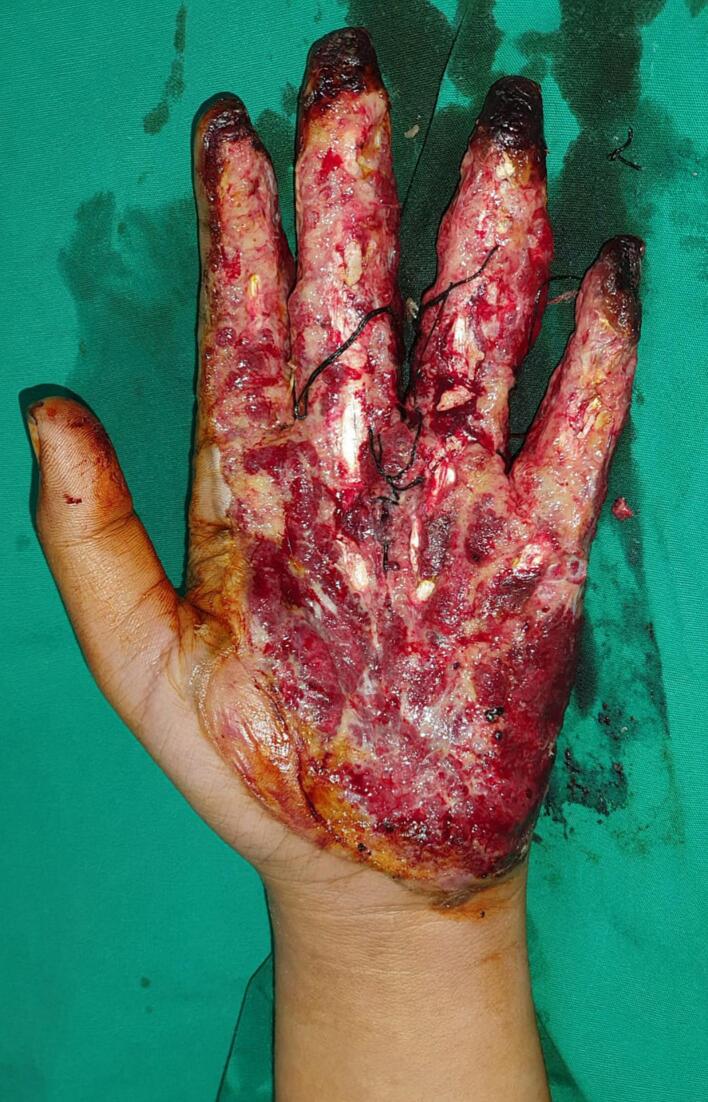
Condition of the hand with fragile blood vessels that were tied with silk before being covered with an abdominal.

For the initial intervention, we performed a comprehensive examination, wound debridement, and escharotomy to release compartment syndrome pressure and improve distal vascularization. Patient got regular burn wound care at the outpatient clinic, the wound bed has shown signs of improvement, but there has been persistent significant bleeding necessitating ligation during treatment. Performing a skin graft on this area would likely result in a low chance of successful graft take due to the recurring uncontrolled bleeding. Given the abundance of fragile blood vessels in the palmar area, it was decided to proceed with immediate wound coverage using a pedicled abdominal flap to preserve the hand (Fig. 2).

**Fig. 3:**
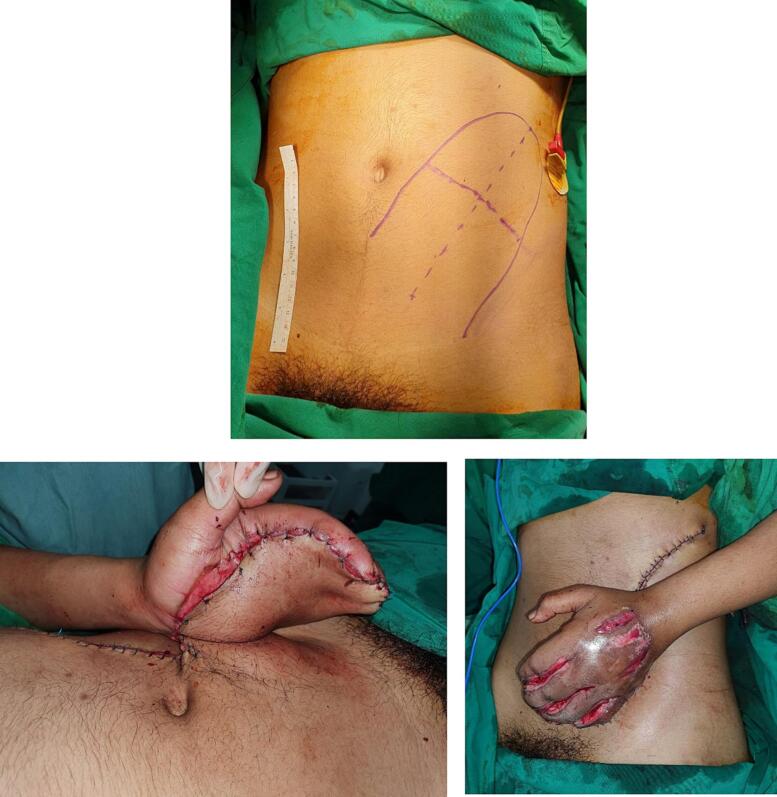
Design of the abdominal flap and the condition immediately after the hand being attached to the abdomen.

**Fig. 4:**
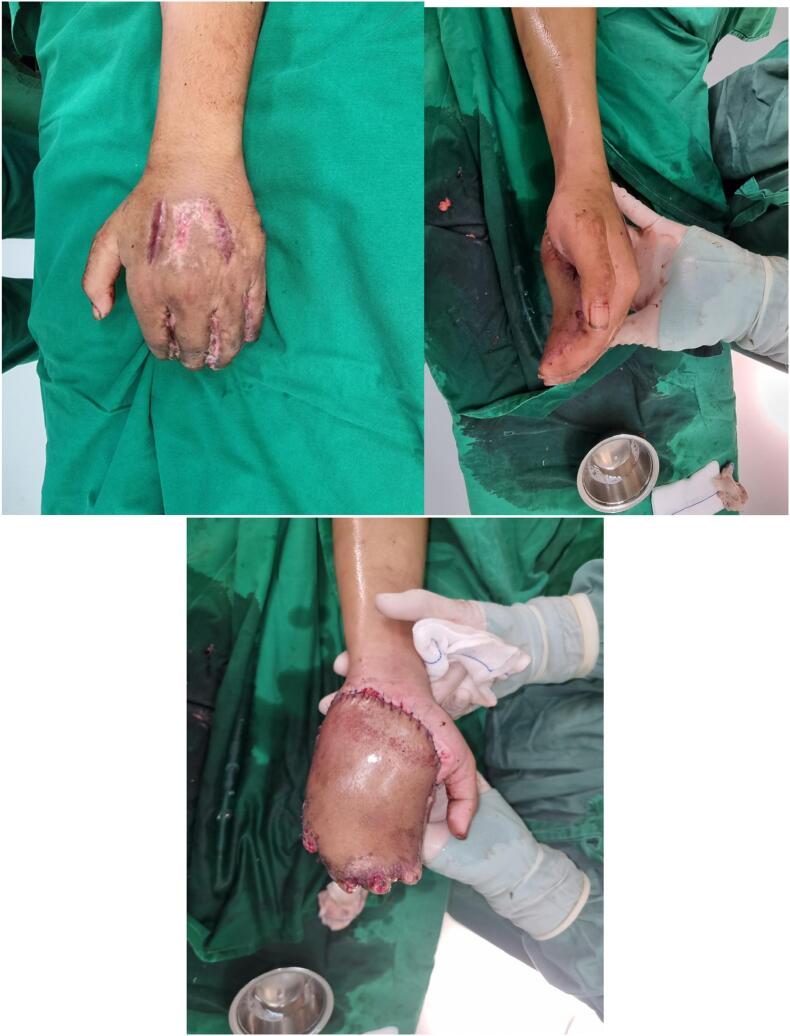
Post pedicled flap division.

For this patient, we created a pedicle based on the superficial circumflex iliac artery (SCIA) vascularized abdominal skin area, and then anchored the skin to the abdominal wall with simple interrupted sutures (Fig. 3). After three weeks, the flap was completely divided as described on Fig. 4. We also performed a necrotomy procedure to remove necrotized tissue from the flap margins. The patient was scheduled to visit the plastic surgery clinic for evaluation of the flap.

**Fig. 5:**
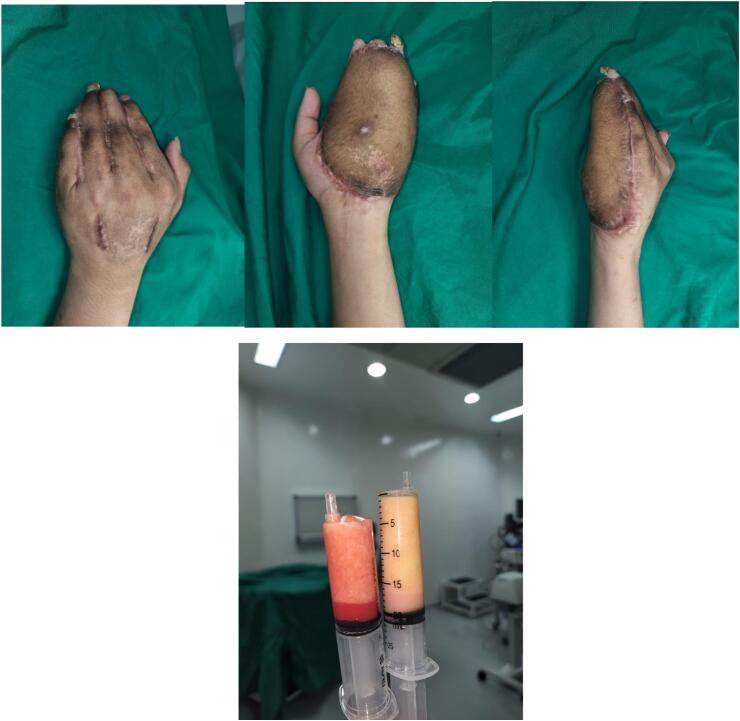
Flap appearances after thinning procedure.

To reach the ideal thickness, a flap thinning procedure was performed three months after the flap separation. Flap thinning involves mechanical type and was performed using the liposuction procedure. During the liposuction procedure, we obtained approximately 40 ml of fat and created a thinner flap as described on Fig. 5.

**Fig. 6:**
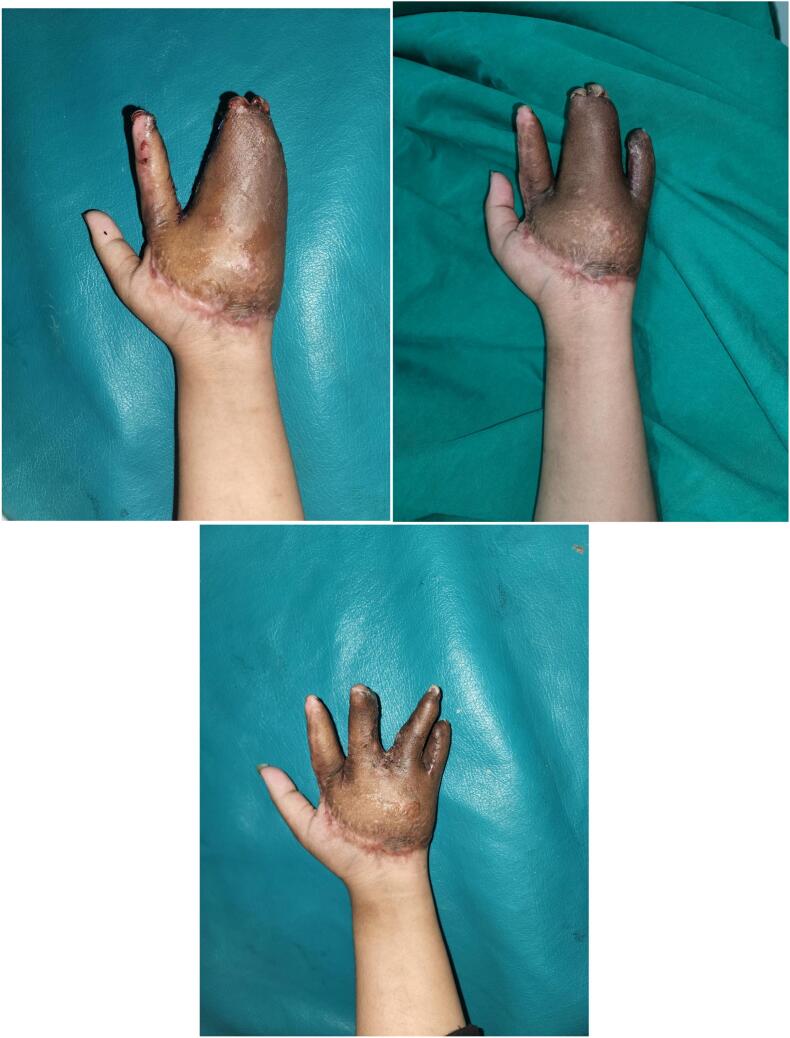
Appearances of 2nd-3rd digits, 4th–5th digits, and 3rd-4th digits (respectively from left to right) after separations procedure.

Since the flap covers the palm and 2nd to 5th digits simultaneously, syndactyly occurs during flap formation. To improve function and aesthetics, we decided to perform digit separation with a longitudinal incision. We separated each pair of fingers within a 21-day timeframe. The separation process occurred sequentially, starting with the 2nd to 3rd digits, followed by the 4th to 5th digits, and finally the 3rd to 4th digits, each at 21-day intervals. The last post-operative results are showed in Fig. 6, nine weeks after the first digit separation surgery.

After the digit separation, patients still have difficulty in performing flexion movements in their fingers. This is due to the deep tissue damage caused by burn trauma. Further action is required for tendon reconstruction to address this issue.

The assessment of hand function impairment was primarily based on patient complaints and basic physical examinations. While we understand the significance of using established functional scales like the MHQ for a more quantifiable and comprehensive evaluation, this approach was not implemented in our study due to several constraints.

The decision to rely on patient complaints and simple physical examination findings was influenced by factors such as the specific context of our patient cohort, resource limitations, and the focus of our study on immediate postoperative outcomes rather than long-term functional assessment.

Patients are also advised to undergo rehabilitation to help restore remaining function and optimize to prevent further contractures. The initiation of hand rehabilitation was strategically planned for two weeks after the surgery for the final digit separation. This timing was primarily based on considerations for optimal wound healing and coincided with the removal of sutures.

We carefully determined that a two-week period post-surgery would allow sufficient time for the initial phases of wound healing to occur, minimizing the risk of disrupting the healing process. Starting rehabilitation too soon after surgery can sometimes lead to complications, such as wound dehiscence or infection, especially in procedures as delicate as digit separation.

The removal of sutures typically signifies a critical milestone in the wound healing process, indicating that the tissue has reached a level of integrity that can withstand the gentle stresses of rehabilitation exercises. At this point, we deemed it safe and beneficial to begin rehabilitation to promote flexibility, strength, and function in the reconstructed hand.

Before the procedure, the patient underwent the process of obtaining informed consent regarding the impending intervention. This step is crucial, especially for the abdominal flap procedure, as it involves the creation of a mass that necessitates full immobilization of the hand until the flap stabilizes. Additionally, the patient was also provided with informed consent regarding potential publication and this work has been reported in line with the SCARE criteria.

## 3 Discussions

We reported hand burned patient due to contact thermal at work. Patient came with full thickness burn at the left palm and compartment syndrome. After the first surgery, the compartment issue has been successfully resolved. A critical part of the initial care of hand burn is to ensure that soft tissues are well perfused. Decompression of high tissue pressure from burn skin with edema is an area in which early surgical intervention can make a tremendous difference to the ultimate outcome. Hands at risk for ischemia include those with circumferential or near-circumferential burns, those with very deep burns, and any electrical injuries involving high or intermediate-range voltage. It is important to look at signs more subtle than the loss of a palpable pulse in named arteries at the wrist [3].

Contact burns result from hot metals, plastic, glass, or hot coals. Although generally small in size, contact burns are challenging in that the injury is often very deep. Burn depth can be predicted based on the temperature of the material and the duration of contact [2]. It was suitable for our case that the patient had a full-thickness burn, reaching deeper tissue, on the palm due to an accident at work. With aggressive wound care and hand therapy, most intermediate-depth palm burns heal in about 2–3 weeks. On the other hand, unoperated deep palm burns heal from the edges with contracture of the palm leading to permanent disability. The decision to perform excision and grafting using thick split-thickness grafts or full-thickness grafts to deep palm burns [2].

Initially, our patient was scheduled for surgery to cover the wound with a skin graft. Following the first surgery, which included debridement and tangential excision, not all eschar could be removed. Therefore, wound care was continued with enzymatic debridement. Dargan in 2021 reported 10-year review of the literature abouy hand burns, Enzymatic debridement results in earlier intervention, more accurate burn assessment, preservation of vital tissue, and fewer skin grafts, and ideally requires regional anesthesia [5]. Repeated handling and debridement of the same contaminated eschar area can also lead to trauma [6]. This serves as an explanation for why we are facing the issue of the patient experiencing bleeding and easily ruptured arteries. So, when we use a skin graft as the choice for wound coverage, bleeding can lead to the formation of a hematoma, which will decrease the vitality of the graft [7].

Pedicle flap is a surgical procedure in which a tissue flap is moved from one area of the body to another while still attached to its original blood supply [7]. The term “pedicle” refers to the blood vessels that connect the flap to its original location. The blood vessels were carefully preserved during the procedure to ensure that the flap remained alive and healthy. Once the flap was moved to its new location, it was repositioned and sutured. Over time, the blood vessels in the flap gradually integrate with the blood vessels in the surrounding tissue, ensuring that the flap remains healthy and functional [8,9]. The surgical procedure of the abdominal pedicle flap in hand reconstruction consists of the following steps:

1. Radical debridement for all selected recipient wounds.
2. After 48–72 h, the wound is covered with a pedicled abdominal flap.
3. The donor pedicle was based on either asingle or combined skin vascularized area of the superficial inferior epigastric (SIEA), superficial circumflex iliac (SCIA), superficial external pudendal (SEPA), and para-umbilical perforator (PUP) arteries.
4. The skin anchored to the abdominal wall with interrupted sutures.
5. At three weeks, the flaps were delayed, and at four weeks the flaps were totally divided.
6. To improve the blood supply and reach the ideal thickness, the flap thinning procedure was performed using mechanical, chemical, or thermal methods.
7. Due to syndactyly formed during flap formation, the digits can be separated to improve function and aesthetics. We divided each pair of fingers within 21 days using longitudinal incisions.

Pedicled abdominal flaps for hand reconstruction were well described by Al-Qattan in 2021. Most of the cases presented involved defects in the dorsum and fingertips. There were two cases with defects in the palmar region, but they did not encompass the entire palmar area as in the patient in this study. According to the paper by Al-Qattan, in selected cases of pedicled abdominal flap reconstruction, implementing active exercises for the attached hand is feasible, safe, and helps minimize the risk of hand stiffness. The results presented showed favorable outcomes [10]. The difference is that there have been no reported cases that specifically address the separation of the abdominal flap in the fingers, as in this study.

In 2022, Putri reported a case of a patient with post-burn contracture. After performing release contracture and scar excision, wound closure was achieved using a glove-like abdominal flap, resulting in favorable outcomes. The main difference from this study lies in the primary location of the defect; this study showcases flap reconstruction in the palmar area, whereas Putri's paper reported flap placement in the dorsal region. Pradier at 2007 also reported hand burns covered by abdominal pocked graft but all of them covered the dorsum area. Both studies yielded positive results in terms of flap viability and the hand's overall condition [11,12].

There are various options for distant pedicle flaps that can be used for hand coverage. The choice of flap depends on the specific soft tissue coverage needed and the experience of the surgeon. One straightforward approach is to apply the Crane Principle. This involves temporarily placing a skin flap with subcutaneous tissue over the wound defect. Later, the skin flap, along with a thin layer of subcutaneous tissue, is returned to the donor site, leaving behind a suitable layer of subcutaneous tissue for skin grafting on the original defect. Other flap procedures involve leaving both skin and subcutaneous tissue attached at the recipient site. Examples of these include the random abdominal wall flap, groin flap, and tensor fascia lata flap, which are explained further below. The cross-arm flap has also been used for immediate coverage of burned hands. The random abdominal wall flap offers the advantage of simplicity, as it does not require specific knowledge of vascular anatomy to perform. It is suitable for covering the entire hand and fingers. However, there are disadvantages to consider. Firstly, the covered hand is not easily accessible for inspection if the patient develops a fever, sometimes necessitating surgical exploration to rule out infection. Secondly, the transplanted skin retains the characteristics of abdominal skin, meaning an increase in body weight will lead to thickening of the transposed abdominal skin and an enlargement of the abdominal area. Lastly, the hand's immobility within the abdominal tissue pocket can result in joint stiffness at the hand, elbow, and shoulder [13].

Following thermal injury, progressive loss of hand function may be a consequence of several factors including direct effects of heat, secondary effects of immobilization, disuse atrophy, soft tissue loss, contracture formation, bacterial wound colonization, decreased circulation, inadequate or inappropriate splinting, or the formation of oedema in connective tissues. Deep burns to the palmar surface of the hand should be monitored carefully. Functional use of the hand and exercise are crucial to maintain ROM [14].

## 4 Conclusions

Thorough examination and appropriate management are important for patients with full–thickness burn injuries. A pedicled abdominal skin flap is one of the best surgical techniques available for full thickness burn injury reconstruction. Through a couple of surgical and rehabilitation procedures, patients with full-thickness burn injuries can regain their closest natural appearance and extremity functions. Our decision to align the start of rehabilitation with the post-suture removal phase was based on a balance between promoting healing and initiating early movement to optimize functional outcomes. We believe that this approach contributed significantly to the positive results observed in our study.

## Ethical approval

Ethical approval for this study (Ethical Committee N° NAC 207) was provided by the Ethical Committee of Dr. Hasan Sadikin General Hospital, Bandung, Indonesia on 15 November 2023.

## Funding

This research did not receive any specific funding.

## CRediT authorship contribution statement

Arif Tri Prasetyo: Study concept or design, data collection, data analysis, interpretation, writing the paper

Lisa Y. Hasibuan: Study concept or design, data collection, data analysis, interpretation, writing the paper

Muhammad Arsyad: Study concept or design, data collection, data analysis, interpretation, writing the paper

## Guarantor

Arif Tri Prasetyo

Lisa Y. Hasibuan

Muhammad Arsyad

## Declaration of competing interest

The authors declare no conflicts of interest related to this study.

## Appendix A Supplementary data

Supplementary video 1 Massive Bleeding due to artery rupture during wound care Supplementary video 1
